# Internalization of stigma among parents of children with autism spectrum disorder in Nigeria: a mixed method study

**DOI:** 10.1186/s40359-021-00687-3

**Published:** 2021-11-21

**Authors:** Aminat Y. Oduyemi, Ifeoma P. Okafor, Ugochukwu T. Eze, Babatunde A. Akodu, Alero A. Roberts

**Affiliations:** 1 Department of Community Health and Primary Care, College of Medicine, University of Lagos, Lagos, Nigeria; 2 Department of Community Health, Lagos University Teaching Hospital, Lagos, Nigeria

**Keywords:** Stigma, Childhood disability, Autism Spectrum Disorder (ASD), Social change, Social inclusion

## Abstract

**Background:**

Autism Spectrum disorder (ASD) has uniquely stigmatizing aspects because children with ASD have no physical markers of their condition. Parents are usually blamed and judgment from others is often internalized (felt stigma).

**Aim:**

This study was conducted to determine knowledge about ASD, negative experiences (enacted stigma), internalization of stigma (felt or self stigma) and its correlates among parents of children with ASD in Lagos, Nigeria.

**Methods:**

This was a cross-sectional study of 230 parents in Lagos, Nigeria employing mixed-method data collection methods. Quantitative data were collected using a structured interviewer-administered questionnaire and analyzed with Epi- Info™ version 7.0 statistical package. Data were summarized with proportions, mean and standard deviation. Chi square and Spearman’s correlation tests were done, and the level of significance was pre-determined at 5% (*p* < 0.05). In-depth interviews were also conducted among six parents to further explore the topic. The interviews were analyzed narratively.

**Results:**

The proportion of mothers and fathers were 175 (76.1%) and 55 (23.9%) respectively. The mean age of respondents was 42 ± 8.5 years. Overall knowledge of ASD was very poor as only 3(1.3%) had good knowledge. Overall, 122(53%) usually had negative experience of parenting a child with ASD (enacted stigma), mothers (17.1%) more than fathers (9.1%). Majority 192(83.5%) internalized stigma. There was a low–moderate correlation between ‘enacted’ stigma and ‘internalized’ stigma (ρ- 0.400, *p* < 0.001). From in-depth interviews, many parents revealed that their child’s condition had negative effects on the family. Many also recounted negative experience of stigma.

**Conclusion:**

Overall, parents of children with ASD had poor knowledge of the condition. Majority internalized stigma and this increases with negative treatment from others. Parents should be properly educated about ASD. Community-based education to increase awareness about ASD in addition to encouraging people to show empathy and reduce stigmatizing behaviour towards parents of children with ASD are recommended.

**Supplementary Information:**

The online version contains supplementary material available at 10.1186/s40359-021-00687-3.

## Introduction

The autism spectrum describes a group of developmental disorders usually most diagnosed in early childhood. Children who, after diagnosis, are construed as being “on the spectrum” have impaired development which manifest before 3 years of age, and abnormal functioning in reciprocal social interaction, communication and restricted, stereotyped, repetitive behaviour [1]. They have language difficulties, lack of initiation of activity and may display antisocial behaviours such as withdrawal, aggression, or disruption, sometimes as a reaction to stress or changes in normal routine [1, 2]. These behaviours constitute a complex formation of stressors which induces tremendous stress on the families, especially the parents resulting in humiliation, social exclusion and isolation [3–6], thereby impacting the mental health of parents and primary carers [7]. These experiences are worse when compared with parents of typically developing children, as well as parents of children with other developmental disabilities [4, 8, 9].

There are persistent challenges with identification, assessment and treatment in Africa which need to be addressed at different levels including community engagement [10]. In Nigeria and other parts of Sub-Saharan Africa (SSA), there is also generally low to average knowledge about ASD among populations with medical/health knowledge [11–14] and presumably lower in the lay population. In other similar settings, caregivers have poor knowledge of the condition [15, 16] influenced by age, education, and ethnicity [16].

Stigma is a very difficult aspect of societal encounter which families with a member with physical or mental challenges experience. It is a concept formed by society and seen as a mark of humiliation, disgrace or guilt [17]. The experience of stigma by parents of children with ASD cuts across cultures globally [2, 16, 18–20]. It is better understood by a distinction between ‘enacted’ stigma and ‘internalized’ (aka self, affiliate or felt) stigma. The former is when a person is treated negatively because of a stigmatizing condition, and the latter is the feeling of shame and embarrassment which comes with such negative treatment and fear of its occurrence [21, 22]. Internalized stigma affects the way a person interprets events around them, thus making them strive to look ‘normal’ [21, 23, 24]. As they internalize stigma, family members and parents of children with disabilities may start experiencing poor self-esteem, increased gloomy emotions, and behaviouraly pull back and hide their feelings of stigmatization from other people [25]. With autism, the stigmatization is unique because the symptoms may be obvious, yet the child’s appearance may not suggest it and also the fact that people know and understand very little about the illness. Parents differ in the way they can respond to the problems brought about by their child’s autistic condition. They tend to adapt better when they understand the condition more, ignore negative reactions from people, distract themselves with work or by spiritual and religious assistance [18, 26]. Perceptions of public stigma are related to parents’ age, gender, intensity of the disability and the child’s age and in social situations, the parents are blamed for bad parenting [6, 25, 27, 28]. Mothers tend to experience stigma more than fathers because historically, there is a natural tendency to blame them for the mis-behaviour of their children, even by their spouses [24, 25, 29].

The types of stigmas experienced by families because of intellectual disabilities including ASD, and how best to reduce this burden constitute major themes for research priorities globally as posited by experts [30]. Previous studies are largely in more advanced countries. The few done in the region are mostly qualitative in nature. There have been calls for action on the topic of ASD in Nigeria where up to 600,000 children may be affected [31]. This study therefore used both quantitative and qualitative methods to assess parents’ knowledge of ASD and explore their experiences encountered in parenting children with autism. It also assessed their internalization of stigma as a result of their child’s diagnosis and negative treatment from others. Findings will add to the limited body of knowledge in the African setting especially in Nigeria and give better direction to alleviating the challenges faced by these parents.

## Methods

Lagos is one of the South Western States in Nigeria. Service provision for children with ASD is dominated by the paediatric services. However, interventions for children with ASD often require parents’ involvement. Several health facilities in the State, both public and private cater to children with ASD. The Child and Adolescent Mental Health Service Centre (CAMHSC) of the Federal Neuropsychiatric Hospital, Yaba, Lagos is the largest child and adolescent mental health service centre in Nigeria [32].

Data were collected using mixed methods. First, a quantitative study was carried out among 230 parents of children with ASD (Additional file 1). The Cochran’s equation (n = z^2^pq/d^2^) for a descriptive cross-sectional study was used to calculate the adequate sample size, z is the standard normal deviate at 95% confidence corresponding to 1.96, ‘d’ is the margin of error desired (0.05) and *p* is the prevalence of the characteristics of the study (proportion of parents who perceived themselves to be stigmatized due to their child’s autistic condition was 84%) [33].

A multistage sampling methodology was used to recruit respondents from public tertiary and general hospitals in Lagos State. The health facilities were selected from a list of health facilities in Lagos State using simple random sampling method and the respondents were selected using consecutive recruitment due to the relatively uncommon nature of the condition and the desire to achieve sample size. Only parents of the children were interviewed, other caregivers were excluded. Data collection was done using semi-structured, pretested, interviewer-administered questionnaire in English, adapted from other studies and Affiliate Stigma Scale that have been used in similar studies in other settings [33–35]. Pretesting was done in another public general hospital which was not selected for the study and slight modifications were made before data collection. Interviewers sometimes used ‘Pidgin English’ as required. The questionnaire elicited information on respondents’ socio-demographic characteristics in the first section: age, relationship to the child, level of education, employment status, number of children, age of child(ren) with ASD. The second section elicited their knowledge of ASD with questions such as ‘What are the symptoms and causes of ASD?’, ‘What is the severity?’, ‘What are the factors associated with ASD?’, ‘When can it manifest? ‘What interventions are available?’ and ‘Is ASD curable?’ The third section assessed their negative experiences as parents of children with ASD. Likert scale of frequency (Never–Always) was used to elicit responses to statements such as ‘People think less of me or my family because of my autistic child(ren)’, ‘An average person is afraid of someone with autism’, ‘I have been stigmatized because of my child(ren)’s condition’, ‘Stigma has affected my family’s ability to make or keep friends’, ‘Stigma has affected my ability to interact with other relatives’, ‘My experiences with stigma have affected my family’s quality of life’. In the last section (fifth), the parents’ internalization of stigma was assessed using a set of statements with responses on a Likert scale of Agreement (Strongly disagree–Strongly agree). Some examples of the statements include: ‘People ignore me or take me less seriously because of my child’s condition’, ‘Negative stereotypes about my child’s condition keep me isolated from social gathering’, ‘Having a child with autism exerts a negative impact on me’, ‘Being around people who do not have a child with autism makes me feel out of place or inadequate’, ‘People without a child with autism could not possibly understand me’ among others.

Qualitative data was collected by in-depth interviews (IIs) to further explore parents’ experiences with raising children with ASD. Participants were selected purposively. Ten parents from the health facilities, who participated initially in the quantitative study were approached face-to-face. Six of them (mothers) agreed to participate in the interview, while the rest (fathers) declined. Data were collected in the clinic on two regular clinic days. Participants were mothers between the ages of 28–41 years, two had post-secondary education, one had secondary education and three had primary education. The IIs lasted for 30 min to one hour and were recorded using a voice recorder in addition to notes taken down during the interviews. A semi-structured interview guide was designed and used to gather more data on areas such as onset of symptoms, diagnosis of child, referral experience, effect of autism on the family, effects on social life and perceived relationship between gender and stigma.

Data were analyzed narratively. Recordings were first transcribed from voice recorder, coded manually by re-reading the transcripts and identifying recurring words or ideas generated from the data.

Quantitative data were analyzed using Epi-Info™ statistical software (Version 7.1.5.2) and summarized using mean, median and standard deviation. The chi-square and students t-test were used to compare differences between selected variables. The level of significance was predetermined at 5% (*p* < 0.05). Correlation analysis was used to quantify the association between selected independent variables (monthly family income, level of education, age of respondents, knowledge of ASD and negative experiences) and self stigma as the dependent variable.

A scoring and grading method was used in the assessment of knowledge, experiences of negative treatment and internalization of stigma. A series of questions on cause, symptoms, interventions for ASD among others were used to assess knowledge. Each correct response was scored one point, otherwise zero. This gave a range of scores from 0 to 16. Percentage score was computed as ratio of respondent’s total score obtainable and a cut-off point of 50% (8) was adopted. Knowledge was graded as good if equal to or greater than 50% and poor if less than 50%. Enacted stigma of the respondents was assessed using 11 statements with responses on a five point Likert scale which were scored as follows: the highest score (5) was assigned to ‘Always’, ‘Often’—4, ‘Sometimes’—3, ‘Rarely’—2 and the lowest score (1) was assigned to ‘Never’. This gave a maximum score of 55 and minimum of 11. The score of 3/5 was used as the cut-off point to grade respondents. Higher scores indicate ‘usually have negative experiences’ (≥ 33) and lower scores (< 33) indicate ‘usually do not have negative experiences.’ Internalization of stigma was assessed using 7 statements with responses on a five point Likert scale of agreement and the scores ranged from Strongly Agree (5) to Strongly Disagree (1). This gave a minimum of 7 and maximum of 35 points. The mid-point score served as the cut-off point to categorize respondents into ‘internalize stigma (≥ 21) and ‘do not internalize stigma’ (< 21).

Prior to interview, the aim and nature of the study were explained to respondents. Participation was purely voluntary and respondents could withdraw from the study at any time. Confidentiality was assured by using anonymous questionnaires. Only numbers and codes were used as a means of identifying the respondents and the questionnaire. The participants were assured that the data collected would be used for research purposes only.

## Results

### Respondents’ socio-demographic characteristics

Two hundred and thirty questionnaires were adequately completed and analyzed. Age of respondents ranged from 20–69 years. The mean age of the respondents was 42.4 ± 8.5 years. Modal age group was 40-49 years. Majority of the respondents were mothers 175 (76.1%), married 209 (90.9%) and Yoruba 166 (72.2%). Most respondents were educated up to secondary school level or higher (88.7%) (Table 1).

**Table 1:** Socio-demographic characteristics of respondents

Variable	Frequency (N = 230)	Percentage (%)
Age (years)		
20–29	17	7.4
30–39	64	27.8
40–49	102	44.4
50–59	44	19.1
60–69	3	1.3
Mean age 42 ± 8.5		
Relationship		
Mother	175	76.1
Father	55	23.9
Marital status		
Married	209	90.9
Single	6	2.6
Widowed/divorced/separated	15	6.5
Tribe		
Yoruba	166	72.2
Hausa	7	3.1
Igbo	53	23.0
Others (Edo, Calabar)	4	1.7
Religion		
Islam	88	38.3
Christianity	142	61.7
Level of education		
No formal education	7	3.0
Primary	19	8.3
Secondary	100	43.5
Post-secondary education	104	45.2

### Family characteristics

A majority 186(80.9%) of the respondents were employed. The modal monthly income/allowance was 40,000–79,000, 133(57.8%) (110–217 USD) using exchange rate at time of study.

Mean number of children was 3** ± **1 SD. Almost all had only one child with ASD and the autistic children were mainly between six to nine years of age (Table 2).

**Table 2:** Family characteristics

Variables	Frequency (N = 230)	Percentage (%)
Employment status		
Employed	186	80.9
Unemployed	44	19.1
Estimated monthly income/allowance of family (Naira)		
10,000–39,000	31	13.5
40,000–79,000	133	57.8
80,000–119,000	47	20.4
120,00–159,000	13	5.7
160,000–200,000	6	2.6
Number of children		
1	12	5.2
2	59	26.7
3	79	34.4
4	61	26.5
5	15	6.5
6	4	1.7
Mean = 3.1 ± 1.1		
Children with autistic disorder		
1	228	99.1
2	2	0.9
Ages of children with autistic disorder (years)		
2–5	51	22.2
6–9	107	46.5
10–13	56	24.4
14–18	16	6.9
Mean = 8.33 ± 3.4		

### Respondents’ knowledge of Autism Spectrum Disorder

The most common sources of information on ASD were health care workers 111(48.3%) and media 73(31.7%). Seventy-three (31.7%) of respondents thought birth complications were the cause of ASD. Many, 126 (54.8%) knew ASD usually manifests in early childhood and that children with ASD are unable to communicate with others, 130(56.5%). Almost half (48.7%) knew economic status of the parents as a factor associated with ASD. A quarter 57 (24.8%) knew ASD cannot be cured. Interventions most mentioned by respondents were behavioural therapy 143 (62.2%) and speech therapy 105(45.7%). Most 227(98.7%) of the respondents had poor knowledge of ASD. The main sources of information on ASD were health workers 111(48.3%) and media 73(31.7%) (Table 3).

**Table 3:** Respondents’ knowledge of Autism Spectrum Disorder

Variable	Frequency (N = 230)	Percentage (%)
*Cause of Autism Spectrum Disorder		
Birth complications	73	31.7
Head injury	38	16.5
Family history	39	16.9
Drinking alcohol in pregnancy	19	8.2
I don’t know	31	13.5
Others	1	0.4
Manifestation of Autism Spectrum Disorder		
Early childhood	126	54.8
Late childhood	71	30.9
Adulthood	1	0.4
I don’t know	32	13.9
*Symptoms of Autism Spectrum Disorder		
Unable to communicate with others	130	56.5
Un-cooperative and being isolated	76	33.0
No eye contact and not close to family member	63	27.4
Language barriers	78	33.9
Talking to themselves or repetitive talking	45	19.6
Narrow life interests and repetitive behaviours	21	9.1
I don’t know	2	0.8
Severity of Autism Spectrum Disorder		
From mild to severe	48	20.9
Usually mild	29	12.6
Usually severe	34	14.8
I don’t know	119	51.8
*Factors associated with Autism Spectrum Disorder		
Economic status of the parents	112	48.7
Educational background of the parents	22	9.5
Age of the parents	30	13.0
I don’t know	46	37.4
Autism Spectrum Disorder can be cured		
Yes	99	43.0
No	57	24.8
I don’t know	74	32.2
*Interventions for Autism Spectrum Disorder		
Behavioural therapy	143	62.2
Speech therapy	105	45.7
I don’t know	33	14.4
Others	3	1.3
*Talents of children with Autism Spectrum Disorder		
Drawing	72	31.0
Singing	57	24.8
Dancing	71	30.8
None	27	11.7
I don’t know	31	13.5
Other talents	1	0.4
Overall knowledge		
Good	3	1.3
Poor	227	98.7
Main source of information		
Family member	22	9.6
Friends	14	6.1
Health care workers	111	48.3
Internet	9	3.9
Media (TV/Radio/Newspaper/Social media)	73	31.7
Others	1	0.4

^*^Multiple responses allowed

### Respondents’ assessment of ‘felt stigma’

Most 92 (40.0%) of the respondents believed that sometimes people think less of those with Autism Spectrum Disorder (ASD), many 111 (48.3%) believed that sometimes an average person is afraid of someone with ASD. One third 77 (33.5%) believed that their experiences with stigma sometimes affect their family’s quality of life. Almost 30% believed that people sometimes discriminate against them because their child’s condition. Overall, 122(53%) of the respondents were classified as ‘usually having negative experience’ of parenting a child with ASD (Table 4).

**Table 4:** Assessment of negative experiences (enacted stigma) among parents of children with Autism Spectrum Disorder

Assessment of felt stigma (N = 230)	Never(%)	Rarely(%)	S/times(%)	Often(%)	Always(%)
People think less of those with ASD	66(28.7)	16(7.0)	92(40.0)	36(15.7)	20(8.7)
People think less of me or my family because of my autistic child(ren)	107(46.5)	23(10.0)	68(29.6)	23(10.0)	9(3.9)
An average person is afraid of someone with autism	44(19.1)	16(7.0)	111(48.3)	37(16.1)	22(9.6)
My child has been stigmatized because of his/her autistic diagnosis	99(43.1)	19(8.3)	63(27.4)	27(11.8)	22(9.6)
I have been stigmatized because of my child’s(ren)’s condition	110(47.8)	17(7.4)	65(28.3)	19(8.3)	19(8.3)
Other members of my family have been stigmatized because of my child’s condition	148(64.4)	20(8.7)	47(20.4)	10(4.4)	5(2.2)
Negative stereotype has affected my family’s ability to make or keep friends	101(43.9)	29(12.6)	73(31.7)	20(8.7)	7(3.0)
Negative stereotype has affected my ability to interact with other relatives	122(53.0)	24(10.4)	59(25.7)	19(8.3)	6(2.6)
My experiences with stigma have affected my family’s quality of life	114(49.5)	14(6.1)	77(33.5)	17(7.4)	8(3.5)
People discriminate against me because I have a child with autism	76(33.0)	105(48.7)	20(8.7)	23(10.0)	6(2.6)
People ignore me or take me less seriously because I have a child with autism	64(27.8)	119(51.7)	21(9.1)	18(7.8)	8(3.5)
Overall experience	Freq(n = 23)	(%)			
Have negative experience	122	53.0			
Do not have negative experience	108	47.0			
Total	230	100.0			

### Respondents’ internalization of stigma (self-stigma)

Seventy-four (32.2%) agreed and 99(43%) strongly agreed to the statement ‘people without a child with ASD could not possibly understand me’ while 54 (23.5%) and 23 (10%) agreed and strongly agreed respectively, that ‘being around people who do not have a child with ASD makes them feel out of place or inadequate.’ A total of 93 (40.5%) agreed/strongly agreed that having a child with ASD exerts a negative impact on them. Most 192(83.5%) respondents self-stigmatize (Table 5).

**Table 5:** Assessment of internalization of stigma (self/felt stigma) among parents of children with Autism Spectrum Disorder

Assessment of stigma (N = 230)	SD (%)	D (%)	N (%)	A (%)	SA (%)
Negative stereotypes about my child’s condition keep me isolated from social gathering	48(20.9)	120(52.2)	23(10.0)	27(11.8)	12(5.2)
I am disappointed in myself for having a child with ASD	105(45.7)	60(26.1)	30(13.0)	25(10.9)	10(4.4)
Being around people who do not have a child with ASD makes me feel out of place or inadequate	79(34.4)	53(23.0)	21(9.2)	54(23.5)	23(10.0)
People without a child with ASD could not possibly understand me	19(8.3)	20(8.7)	18(7.8)	74(32.2)	99(43.0)
Having a child with ASD exerts a negative impact on me	43(18.7)	64(27.8)	30(13.0)	48(20.9)	45(19.6)
Having a child with ASD makes me think I am less important to others	99(43.0)	67(29.1)	26(11.3)	23(10.0)	15(6.5)
Nobody would be interested in getting close to me because I have a child with ASD	119(51.7)	64(27.8)	20(8.7)	14(6.1)	13(5.7)
Overall assessment	Freq(N = 230)	(%)			
Self-stigmatize	192	83.5			
Do not self-stigmatize	38	16.5			
Total	230	100.0			

*SA* strongly agree; *A* agree; *N* neutral; *SD* strongly disagree; *D* disagree

### Factors associated with experience of stigma

There was a statistically significant association between level of education (*p* = 0.001), monthly family income (*p* < 0.001) and enacted stigma. Respondents reporting the lowest family income had highest rate of enacted stigma (*p* < 0.001). Same goes for self-stigma but the difference was not statistically significant (*p* = 0.095) (Table 6).

**Table 6:** Test of association between enacted and self-stigma, and socio-demographic variables using Chi Squared test

	Yes(%)	No(%)	Total(%)	*X*^2^	*p* value
	Enacted stigma				
Level of education					
Post-secondary	39(39.0)	61(61.0)	100(100)	14.08	0.001
Secondary	67(64.4)	37(35.6)	104(100)		
Primary/no formal	16(61.5)	10(38.5)	26(100)		
Monthly family income					
10,000–39,000	27(87.1)	4(12.9)	31(100)	23.25	< 0.001
40,000–79,000	55(41.4)	78(58.6)	133(100)		
80,000–200,000	40(60.6)	26(39.4)	66(100)		
Relationship to child					
Mother	96(54.9)	79(45.1)	175(100)	0.97	0.355
Father	26(47.3)	29(52.7)	55(100)		
	Self stigma				
Age					
20–39	64(79.0)	17(20.9)	81(100)		
40–49	87(85.3)	15(14.7)	102(100)	1.90	0.388
50–69	41(87.2)	6(12.8)	47(100)		
Level of education					
Post-secondary	87(87.0)	13(13.0)	100(100)		
Secondary	83(79.8)	21(20.2)	104(100)	1.94	0.379
Primary/no formal	22(84.6)	4(15.4)	26(100)		
Monthly family income					
10,000–39,000	28(90.3)	3(9.7)	31(100)		
40,000–79,000	105(78.9)	28(21.1)	133(100)		
80,000–200,000	59(89.4)	7(10.6)	66(100)	4.71	0.095
Religion					
Christianity	118(83.1)	24(16.9)	142(100)		
Islam	74(84.1)	14(15.9)	88(100)	0.04	1.000

### The relationship between ‘negative experiences/treatment’ and ‘internalization of stigma’

There was a low-moderate positive correlation between experience of stigmatization and self-stigmatization (ρ 0.400). The more the negative experience (enacted stigma), the higher the level of internalization (self-stigma) (Table 7).

**Table 7:** Spearman’s correlation matrix of the self-stigma score, enacted stigma score, knowledge score and socio-demographic variables

Variables					
	Negative experience (enacted stigma)	Knowledge of Autism Spectrum Disorder	Age of respondents	Level of education	Monthly family income
Self- stigmatization ρ (rho)	0.400	0.048	− 0.015	− 0.013	− 0.058
*p*-value	< 0.001	0.468	0.825	0.845	0.038

ρ Spearman correlation coefficient

### In-depth interview results

#### Onset of symptoms

Most of the parents noticed their child was a little different at age three. As one mother commented: “*He was not really acting like his elder brother when he was at that age, his elder brother was calm and playful but he was troublesome and withdrawn from people*” (35, Trader, Primary level of education).

#### Referral experience

Referral experience differs from one parent to another. Most parents were referred from the hospital where the child was delivered. However, parents who gave birth to their child at home sought other means. As one mother commented: *“When I noticed his behaviour was a little different, I told people around me about his behaviour and I was told to take him to the hospital”* (41, Hairdresser, Primary level of education). Prior to this, majority of the parents have had contacts with other parents of children with ASD, though not relatives.

#### Diagnosis of child’s condition

Parents confessed feeling sad and described the period the child was diagnosed with ASD as a tough time. As one commented: *“It was really bad, it was a very tough time for me, I have been seeing children like that and I always pray that I would never have such a child, but I have accepted my fate”* (35, Trader, Primary level of education).

However, some did not feel sad but were optimistic about the child’s condition. As one mother said: *“I don’t feel bad at all, because I believe he will be okay by the grace of God”* (28, Fashion designer, Secondary level of education).

#### Nature of child’s presenting symptoms

The symptoms of ASD vary from one child to another and depend on the position the child is on the spectrum. Some children can be a bit aggressive while others can be calm. Majority of the parents described the child as being aggressive and expressed their frustration with this. As one mother lamented: *“She just suddenly starts to scream and hit her head on the floor and I just wonder what is wrong with her”* (33, Civil Servant, Post-secondary level of education). However not all children are aggressive. In the words of one mother: *“the only thing he does is that when you are talking to him, he does not pay attention and he is always laughing when you are talking to him”* (41, Hairdresser, Primary level of education).

#### Effect of autism on family

Effect of autism on the family varied from one family to another. It has affected the family life balance with negative consequences. For a few of them, it appeared not to be bad. One mother commented: *“it does not affect my family at all”*. But others lamented that the time dedicated to the care of the affected child(ren) has impacted family life negatively. One mother said: *“I spend most of my time trying to look after him and I don’t have time to look after his siblings”* (38, Teacher, Post-secondary level of education). According to another mother: *“when we have visitors come around and he starts to display his behaviours and I find it difficult to keep him calm, I feel very ashamed because visitors do not know about his condition and they think that he acts that way because we spoilt him. Sometimes I blame myself for giving birth to him”.* (35, Trader, Primary level of education).

#### Social experience

Majority of the parents interviewed feel embarrassed and rejected when they go out with their autistic child. As one mother complained: “*When we go out and he starts to scream, people around us just stop to stare at us………, I feel very embarrassed”* (36, Trader, Primary level of education) Another mother also said: *“when I take him out, people leave where we are to move another section, and also segregate him from other children. It is really embarrassing and makes me feel rejected”* (38, Teacher, Post-secondary level of education).

Another parent’s view: *“if people like you, they will like your child no matter the condition of the child but if they don’t like you, they will not like your child”* (28, Fashion designer, Secondary level of education).

#### Relationship between parent’s gender and ‘felt’ stigma

Most of the parents believed that social experience of stigma has nothing to do with gender of the parents but depends on the child’s behaviour. As one mother reported: *“it does not matter who he goes out with, however his father does not like taking him out”* (41, Hairdresser, Primary level of education).

## Discussion

ASD stigma not only affects the children but also their parents. Parents internalize stigma as a result of experiencing stigma through association with their children with ASD. This study revealed very poor knowledge of ASD among parents. Self-stigma increases significantly with negative experiences from others.

The mean age of the respondents was similar to that of respondents in Ethiopia and Hongkong where the mean ages were 45.9 and 42.5 years [19, 25]. Majority of the respondents in this study were mothers, married, employed and had post-secondary level of education. Other studies also comprised mostly mothers [16, 19, 26, 36, 37].

Sources of information on ASD were mainly from health care workers and media (TV/Radio/Newspaper/Social media). The study was facility-based and thus respondents would have relied on health workers for information on ASD as people generally know very little about the condition. Other sources of information on ASD include family and friends and rarely the internet. This is similar to another group of respondents in Addis Ababa, Ethiopia where main sources of information were health extension workers (52.9%) [20]. Informal sources of information on the condition are also common in Africa [38], but in USA, main sources of information include internet (73%), books, magazines or video tapes (71%) and other parents of children with ASD (42%) [35]. Though our respondents were educated, this variation may be due to cultural and spatial differences as internet facilities are better in the developed countries.

Only very few of our respondents had good knowledge about ASD, a reflection of the lack of proper education on the condition by the health workers since the study was facility-based. The role of informal sources of information and cultural beliefs should be considered. The poor knowledge recorded supports reports from other authors in LMIC [15, 16, 36]. This finding is not surprising as health workers have also shown low awareness/knowledge [39]. Conversely, studies among teachers in USA and Pakistan showed better knowledge [40, 41], and in Zambia, mothers of children with epilepsy were considered to have high knowledge about the condition [42].

Regarding the symptoms of ASD and interventions such as behavioural and speech therapy respondents had good knowledge because they have first-hand experience of these symptoms in their children and they were recruited from facilities where such interventions were likely available. Also in the Zambian study on epilepsy, mothers lacked knowledge in the domain of ‘cause’ of the condition [42]. Cultural beliefs can also shape the understanding of ASD even among immigrants in highly developed countries [43]. So far, socio-demographic factors influencing knowledge have largely not been reported specifically among parents and caregivers of children with ASD. The population-based study on caregivers of young children (not specific for ASD) in Nepal, found that knowledge was significantly better among the older and more educated respondents and those from the upper caste/ethnic group [15].

Overall, more than half of the respondents reported negative experiences (enacted stigma) in parenting a child with ASD. This is higher than rates reported in North Carolina, USA where the study compared both Japanese and American mothers’ experience of stigma. Only 28.1% and 22.2% of both Japanese and American mothers had experienced social stigma and rejection respectively [44]. Cultural variations likely account for this differential. Most parents in Africa report experiences of enacted stigma according to studies which were largely qualitative with fewer respondents [18–20, 26]. Stigma is not influenced by the parents’ or child’s personal characteristics (except for religion) but increased by other attributes such as having sought traditional help and provided supernatural explanation for their child’s condition [20].

In the in-depth interviews, many parents recounted their negative experiences. These themes resonate across other similar studies across countries [16, 18, 24–27, 35]. Our respondents equally lamented the negative effects of ASD on their family life which also corresponds with reports from the afore-mentioned studies. Amidst all the difficulties, stress, and negative experiences from parenting children with ASD, parents still maintain a positive outlook to life [45].

In our study, mothers had more negative experience of parenting a child with ASD compared to the fathers though the difference was not statistically significant. Also in Iran, mothers (58.6%) had more negative experience than the fathers (35.6%) [46]. This is likely related to the fact that mothers are usually the primary caregivers of the child and are responsible for the child. But there might be other explanations for this because some children are afraid of their fathers and so they respond to their commands to behave [15]. Only mothers participated in the in-depth interviews. Perhaps the fathers who declined might have given us a different narrative.

Findings from our study reveal that majority internalized stigma, higher than the rates reported by other authors. For example, among Chinese, results indicated that about half (50.9%) internalized stigma [28] and also about half in Virginia [35]. In Israel, it was reported to be low [9] but in Hong Kong, internalized stigma was severe among respondents [25]. Self-stigmatization of respondents was said to be prevalent because respondents showed low self-esteem, high shame proneness and poor family adaptability [6, 25]. This is because parents, who have utilized coping strategies such as meditation, research on autism, and prayer experienced less negative emotions. Many parents in our study experienced enacted stigma and so it is not surprising that they feel ashamed and internalize this stigma. One of the in-depth interviewees confessed that she blames herself for giving birth to her autistic child. This is an expression of a very strong negative emotion.

Parents who were less educated and families with lower monthly income experienced more negative experiences with a significant difference. As it were, their low socio-economic conditions already expose them to stigma and negative experiences generally. Respondents at the lower and upper family income groups experienced more ‘self stigma’ with no significant difference. Again, there are few quantitative studies reporting on the personal variables influencing self-stigma and most of the variables such as caregivers’ gender, age, educational level, children’s ages were insignificant [27, 28].

Further analysis showed a low-moderate positive correlation between negative experiences with parenting a child with ASD and internalization of stigma. This means that other people make them feel bad about themselves due to their children’s condition. The Virginia study showed statistically significant difference between respondents’ experience of parenting a child with ASD and assessment of self-stigma [35]. Other factors which enhance internalized stigma among parents of children with developmental disorders include poor family/social support, non-acceptance of the condition, low awareness/knowledge of the condition and psychological distress [19, 47]. Proposed interventions need to address the issue of stigmatization in the larger society and reduce internalization of stigma among parents. During the in-depth interviews, some parents confessed that they had ‘accepted their fate’, or that they had ‘left everything to God’. Their responses are influenced by cultural and religious inclinations which supports them not to give up in such situations. Parents usually adopt various coping strategies such as religion/spirituality, respite care, talking to relatives and friends, acceptance, and self-compassion among others [9, 16, 18, 20, 26, 47, 48]. Various interventions to promote wellbeing in parents of children with ASD have been done around the world but hardly in developing countries. Some involved spirituality [49] or multi component involving psycho education, cognitive restructuring strategies and compassion focused methods to help parents cope with, prevent, and reduce the harmful effects of self-stigma and eventually improve their mental health [50]. Varying degrees of success were recorded with implications for scaling.

### Strengths and limitations of study

Findings from this study contributes to the information base on this topical issue especially in the developing countries where mental health services are grossly inadequate. The use of mixed-method approach increased the body of knowledge and rigorous data collection processes speaks to the scientific quality.

Being a cross-sectional study, causality cannot be inferred. Consecutive recruitment of respondents could also have some ‘clustering’ effect. Likert statements assessing stigma were phrased one-directional thereby increasing chances of response-set bias. In addition, the generalizability of findings may be limited. Further studies with a community component should be carried out to understand the issues surrounding ‘enacted’ and ‘self’ stigma.

## Conclusion

The overall knowledge on ASD among respondents was poor and their main source of information was health workers. In-depth interviews showed that many parents were embarrassed by their child’s behavior. Parents who were less educated and from poorer families had significantly more negative experience than their counterparts. The stigmatization that the parents experienced from others contributed significantly to internalizing their stigma. There is need for interventions in the larger society, encouraging people to show empathy and reduce stigma. Counselling services to overcome internalization of stigma would also be beneficial. Issues surrounding internalization of stigma need further exploration at the community level.

## Supplementary Information

**Additional file 1.** Questionnaire.

## Data Availability

The datasets used and/or analyzed during the study are available from the corresponding author on reasonable request.

## Abbreviations

ASD: Autism Spectrum Disorder
CAMHSC: Child and Adolescent Mental Health Service Centre
II: In-depth Interview
