# Supplementary material for: Internalization of stigma among parents of children with autism spectrum disorder in Nigeria: a mixed method study
Source: BMC Psychol. 2021 Nov 21;9:182. doi: 10.1186/s40359-021-00687-3 (PMC8607563; doi:10.1186/s40359-021-00687-3)
Supplement: Supplementary file 1 — Additional file 1. Questionnaire. [file 40359_2021_687_MOESM1_ESM.docx]

**QUESTIONNAIRE**

**Internalization of stigma among parents of children with autism spectrum disorder in Nigeria: a mixed method study**

**SECTION A: SOCIO-DEMOGRAPHIC INFORMATION**

**Respondent: Parent** (Father/Mother)

1. Age (as at last birthday) ……………...
2. Relationship a) Mother [ ] b) Father[ ]
3. Marital Status a) Single [ ] b) Married [ ] c) Widow, Divorced, Separated [ ]
4. Tribe a) Yoruba [ ] b) Hausa [ ] c) Igbo [ ] d) Others (Please specify) ……….............
5. Religion a) Christianity [ ] b) Islam [ ]
6. Level of Education a) No formal education [ ] b) Primary [ ] c) Secondary [ ] d) Post-Secondary Education [ ]
7. Employment status a) Employed [ ] b) Unemployed [ ]
8. Estimated monthly income/allowance ........................
9. How many children do you have? .............
10. How many children do you have with autistic disorder? .............
11. What is/are the age(s) of your child(ren) with autistic disorder? ..............

**SECTION B: KNOWLEGE OF AUTISM SPECTRUM DISORDER**

1. Where did you hear about ASD? a) Health Care Workers [ ] b) Family member [ ] c) Media (TV/Radio/Newspaper/Social media) [ ] d)Friends [ ] e) Internet [ ] f) Others(Please specify) ……………………………….
2. What are the symptoms of the ASD? a) Unable to communicate with others [ ] b) un-cooperative and being isolated [ ] c) No eye contact and not close to family members[ ] d) Language barriers [ ] e) Talking to themselves or repetitive talking [ ] f) Narrow life interests and repetitive behaviours [ ] g) I don’t know [ ] h) Others (Please specify)..............
3. What is the cause of ASD? a) Birth complications [ ] b) Head injury [ ] c) Family history [ ] d) Drinking alcohol in pregnancy [ ] e) Exact cause unknown f)I don’t know [ ] f) Others (Please specify)..............
4. What can be done for children with ASD? a) Behavioural therapy [ ] b) speech therapy [ ] c) I don’t know [ ] d) Others (Please specify)...................
5. What is the severity of ASD? a) From mild to severe [ ] b) Usually mild [ ] c) Usually severe [ ] d) I don’t know [ ]
6. When can ASD manifest? a) early childhood [ ] b) late childhood [ ] c) adulthood[ ] d) I don’t know [ ]
7. Is ASD curable? a) Yes [ ] b) No [ ] c) I don’t know [ ]
8. What factors are associated with ASD? (You can tick more than one box)

a) Economic status of the parents [ ] b) Educational background of the parents [ ] c) Age of the parents [ ] d) I don’t know [ ]

1. What types of talent do children with ASD have? a) Drawing [ ] b) Singing [ ] c) Dancing [ ] d) None [ ] e) I don’t know [ ] f) Others (Please specify)..............

**SECTION C: EXPERIENCES OF PARENTING A CHILD WITH AUTISM**

**Please tick the box corresponding to the response**

|  | **NEVER** | **RARELY** | **SOMETIMES** | **OFTEN** | **ALWAYS** |
| --- | --- | --- | --- | --- | --- |
| People think less of those with autism |  |  |  |  |  |
| People think less of me or my family because of my autistic child(ren) |  |  |  |  |  |
| An average person is afraid of someone with autism |  |  |  |  |  |
| My child(ren) has been stigmatized because of his/her autism |  |  |  |  |  |
| I have been stigmatized because of your child(ren) condition |  |  |  |  |  |
| Other members of my family have been stigmatized because of my child(ren)’s condition |  |  |  |  |  |
| Stigma has affected my family’s ability to make or keep friends |  |  |  |  |  |
| Stigma has affected my ability to interact with other relatives |  |  |  |  |  |
| My experiences with stigma have affected my family’s quality of life |  |  |  |  |  |

**SECTION D: ASSESSMENT OF SELF-STIGMA**

**Please tick the box corresponding to the response**

Use the following scale to rate your answers; **SD -STRONGLY DISAGREE,**

**D - DISAGREE, N - NEUTRAL, A - AGREE, SA-STRONGLY AGREE**

|  | SD | D | N | A | SA |
| --- | --- | --- | --- | --- | --- |
| People discriminate against me because I have a child with autism |  |  |  |  |  |
| People ignore me or take me less seriously because of my child’s condition |  |  |  |  |  |
| Negative stereotypes about my child’s condition keep me isolated from social gathering |  |  |  |  |  |
| I am disappointed in myself for having a child with autism |  |  |  |  |  |
| Being around people who do not have a child with autism makes me feel out of place or inadequate |  |  |  |  |  |
| People without a child with autism could not possibly understand me |  |  |  |  |  |
| Having a child with autism exerts a negative impact on me |  |  |  |  |  |
| Having a child with autism makes me think I am less important to others. |  |  |  |  |  |
| Nobody would be interested in getting close to me because I have a child with autism |  |  |  |  |  |

**IN-DEPTH INTERVIEW GUIDE**

1. At what age did you notice that your child may not be like other children?
2. What did you do first when you noticed that your child may not be like other children?
3. How did you get here (ie to the Health Facility)?
4. Did you know anybody that had an autistic child before you had yours? (probe if they were family members)
5. How did you feel when your child was diagnosed with ASD?
6. What behaviours do your child exhibit? Is he/she aggressive?
7. How would you say the present condition of your child has affected/affect your family?
8. Do people treat you or your family members differently because of your child’s condition?
9. What kind of behaviour does your child exhibit when you go out with your child?
10. Do you think that if the child goes out with the other parent, (s)he would experience the same treatment?
